# Recalibrating the evidence level in hepatocellular carcinoma: Integrating efficacy and effectiveness of immunotherapy

**DOI:** 10.1016/j.iliver.2025.100200

**Published:** 2025-11-06

**Authors:** Han Wu, Xin-Fei Xu, Jia-Hao Law, Tian Yang

**Affiliations:** Department of Hepatobiliary Surgery, Eastern Hepatobiliary Surgery Hospital, Naval Medical University, Shanghai 200438, China; Division of Hepatobiliary and Pancreatic Surgery, Department of Surgery, National University of Hospital, Singapore 119074, Singapore; Department of Hepatobiliary Surgery, Eastern Hepatobiliary Surgery Hospital, Naval Medical University, Shanghai 200438, China

The central challenge in advancing hepatocellular carcinoma (HCC) therapy no longer lies solely in demonstrating efficacy under ideal trial conditions, but in ensuring that these findings translate into meaningful improvements in outcomes for the complex patients we treat in real-world practice.^1^ As immunotherapy leads to increasingly complex survival curve patterns including delayed separation and crossing hazards, the exploration of advanced analytical techniques such as the MaxCombo test and restricted mean survival time (RMST) has gained considerable attention.^2^ While such methodological rigor is essential for accurately interpreting trial results, it also prompts a broader reflection for the oncology community: in our pursuit of statistical sophistication, are we inadvertently widening the gap between trial efficacy and real-world effectiveness? The distinction between efficacy, the performance of an intervention under ideal and controlled conditions, and effectiveness, its impact in the complex and varied real-world setting, has never been more critical to address.

For decades, the randomized controlled trial (RCT) has been regarded as the gold standard of evidence. Its strength lies in its internal validity, achieved through strict protocols, selective inclusion criteria, and controlled settings. Yet, this strength is also its primary limitation in the context of HCC, a disease marked by profound biological and clinical heterogeneity. Patients enrolled in pivotal RCTs often represent only a narrow subset of those encountered in clinical practice, while individuals with preserved liver function, good performance status, and limited comorbidities. This creates a therapeutic ideal that seldom aligns with the realities of global HCC burden, where diversity in etiology, liver reserve, and access to care profoundly influence outcomes. The recent experience from the IMbrave050 trial illustrates this dissonance.^3^^,^^4^ While the study initially met its primary endpoint of recurrence-free survival, longer follow-up revealed a diminishing statistical significance. This shift does not reflect a failure in trial execution but rather highlights the fragility of conclusions drawn from a narrowly defined population and the potential volatility of surrogate endpoints when extrapolated to a real-world practice.

The advancement of statistical methodologies is undeniably valuable for unpacking complex time-to-event data. However, this very sophistication introduces two significant challenges. First, it creates a translational gap between statistical findings and clinical communication. Metrics such as the difference in RMST or the interpretation of crossing hazard functions, while meaningful to methodologies, are difficult to translate into the simple, patient-centered dialogue required at the bedside. For a patient facing a life-limiting illness, the central questions remain: "Will this treatment give me more meaningful time, and will that time be of good quality". A marginal survival advantage, identified through sophisticated modeling, may represent a statistical success but hold little clinical relevance for a population whose median survival remains desperately short. Second, there is a risk that complex methods are used to extract marginal signals from datasets, potentially giving the impression of a benefit where none of substantial clinical value exists. The primary role of statistical analysis should be to clarify the clinical message, not to obscure the absence of a robust treatment effect with technical complexity.

Traditionally, the true paradigm shifts in HCC therapy have not been driven by statistical novelty but by treatments that delivered unequivocal and clinically palpable benefits. The introduction of sorafenib established a new standard not because of an elegant *p*-value, but because it demonstrably extended overall survival in a broad population of advanced HCC patients.^5^^,^^6^ Similarly, the subsequent revolutions brought by immune checkpoint inhibitor combinations, such as atezolizumab plus bevacizumab, redefined care because their benefits were clear, substantial, and subsequently corroborated across diverse real-world cohorts. Their impact was intuitively understood by clinicians because it translated into visible patient outcomes. In contrast, therapies that only demonstrate incremental hazard reductions in highly selective trial populations risk promoting an optimism that may not materialize in everyday practice, potentially leading to the adoption of treatments with limited patient-level value.

The immunotherapy era in hepatology calls for a deliberate recalibration of how we evaluate evidence.^7^^,^^8^ We must foster an ecosystem where the traditional RCT is complemented, not replaced, by other vital forms of evidence. This includes a greater emphasis on pragmatic trials and large-scale registries, which capture data from patients who mirror real-world heterogeneity and provide indispensable insights into long-term safety, treatment sequencing, and effectiveness in underrepresented groups. Furthermore, patient-reported outcomes and quality-of-life metrics should be elevated from secondary considerations to co-primary endpoints in many trials, ensuring that we evaluate not just the quantity of life gained but its quality. Finally, the systematic synthesis of real-world evidence is crucial for understanding patterns of care, cost-effectiveness, and accessibility, while these questions that RCTs are inherently unable to answer. This integrated approach creates a virtuous cycle, where real-world observations inform trial design, and trial results are continuously validated and contextualized in clinical practice.

In conclusion, while methodological refinement is a hallmark of scientific progress, it must serve the clinical meaning of research, not overshadow it. As we develop and embrace more complex analytical tools, we must remain vigilant that our focus on *p*-values and hazard ratios does not detach us from the human experience of the disease we aim to treat.^9^ The ultimate validation of a therapy's worth lies not in its statistical performance within a curated cohort, but in its consistent and meaningful ability to improve survival and quality of life for the diverse spectrum of patients we serve. By recalibrating our evidence hierarchies to accord equal weight to real-world effectiveness, we can ensure that the pursuit of scientific innovation remains firmly anchored in the principles of patient-centered care.

## CRediT authorship contribution statement

**Han Wu:** Investigation, Methodology, Writing – original draft. **Xin-Fei Xu:** Data curation, Methodology, Writing – review & editing. **Jia-Hao Law:** Validation, Writing – review & editing. **Tian Yang:** Conceptualization, Funding acquisition, Investigation, Methodology, Writing – original draft, Writing – review & editing.

## Informed consent

Not applicable.

## Ethics statement

Not applicable.

## Data availability statement

Not applicable.

## Declaration of generative AI and AI-assisted technologies in the writing process

During the preparation of this work the authors did not use generative AI or AI -assisted technologies.

## Funding

None.

## Declaration of competing interest

All authors have declared no conflict of interest. Tian Yang is Executive Editor of the journal, and he was not involved the editorial review or the decision to publish this article.
